# Functional Outcomes of Anatomic Anterior Cruciate Ligament Reconstruction Using Retrograde Femoral Socket Drilling via a Far Anteromedial Portal Combined with a Conventional Antegrade Tibial Tunnel: A Retrospective Cohort Study

**DOI:** 10.3390/jcm15103651

**Published:** 2026-05-09

**Authors:** Harun Köse, Ekrem Özdemir, Enes Gündüz, Hakan Ertem, Hüseyin Utku Özdeş, Okan Aslantürk, Emre Ergen

**Affiliations:** 1 Department of Orthopedics and Traumatology, Prof. Dr İlhanÖzdemir State Hospital, 28200 Giresun, Turkey; harunkose925@gmail.com; 2 Department of Orthopedics and Traumatology, Erzurum City Hospital, 25240 Erzurum, Turkey; e.o.1986@hotmail.com; 3 Department of Orthopedics and Traumatology, Battalgazi State Hospital, 44280 Malatya, Turkey; enesgunduz1907@gmail.com; 4 Department of Orthopedics and Traumatology, İnönü University, 44280 Malatya, Turkey; hakan.ertem@hotmail.com (H.E.); okaslanturk@hotmail.com (O.A.); emre.ergen@inonu.edu.tr (E.E.)

**Keywords:** anterior cruciate ligament, ACL reconstruction, retrograde femoral socket drilling, far anteromedial portal, hamstring autograft, functional outcomes, Tegner-Lysholm score, IKDC, Cincinnati score, epiligament

## Abstract

**Background/Objectives**: Anterior cruciate ligament (ACL) reconstruction remains the accepted standard of care for ACL ruptures in physically active individuals. Various surgical techniques have been developed to achieve anatomic reconstruction and optimize functional outcomes. The aim of this study was to descriptively report the early functional outcomes of anatomic ACL reconstruction performed using retrograde femoral socket drilling using a FlipCutter through a far anteromedial portal combined with a conventional antegrade tibial tunnel, without claiming superiority over alternative techniques. **Methods**: This single-center, single-arm retrospective cohort study included 33 consecutive male patients with ACL rupture who underwent arthroscopic ACL reconstruction using hamstring tendon autograft between 2021 and 2022 at a tertiary academic medical center. The surgical technique employed retrograde drilling of the femoral socket using a FlipCutter device introduced through a far anteromedial portal; the tibial tunnel was created with a standard outside-in aiming guide. The pre-specified primary outcome was the change in the Tegner–Lysholm score from baseline to 12 months; secondary outcomes were the Modified Cincinnati and International Knee Documentation Committee (IKDC) subjective scores and clinical stability tests. Functional outcomes were assessed preoperatively and at 6 weeks, 3 months, and 12 months postoperatively using repeated-measures testing with the Friedman test and post hoc Wilcoxon signed-rank tests with Bonferroni correction. Reporting followed the STROBE recommendations for observational studies. **Results**: All 33 patients (100% male) completed the 12-month follow-up. The mean age was 28 years (range: 18–44), and sports-related injuries accounted for 84.8% of cases. Significant improvements were observed in all functional scores from preoperative to 12-month postoperative assessments (*p* < 0.001). The mean Tegner-Lysholm score improved from 46.8 ± 17.3 preoperatively to 83.7 ± 10.5 at 12 months (mean change +36.9, 95% CI 30.3 to 43.5; matched-pairs effect size r = 0.87). The mean IKDC score increased from 36.3 ± 14.4 to 68.4 ± 15.1 (mean change +32.1, 95% CI 25.3 to 38.9; r = 0.84), and the Cincinnati score improved from 41.3 ± 15.9 to 80.2 ± 10.9 (mean change +38.9, 95% CI 32.6 to 45.2; r = 0.86). All observed mean changes exceeded the minimal clinically important difference (MCID) reported for these instruments in ACL populations. Postoperative stability assessment demonstrated restoration of knee stability in the majority of patients, with 66.7% showing a negative anterior drawer test at final follow-up. **Conclusions**: Anatomic ACL reconstruction utilizing retrograde femoral socket drilling using a FlipCutter through a far anteromedial portal combined with a conventional antegrade tibial tunnel was associated with satisfactory early functional outcomes in a small, all-male cohort, comparable to those reported for contemporary anatomic ACL reconstruction techniques. Given the retrospective, single-arm design, modest sample size, homogeneous all-male cohort, absence of instrumented laxity or return-to-sport data, and absence of multivariable adjustment, any suggestion of technique-specific advantages should be regarded as hypothesis-generating. Comparative effectiveness against other anatomic techniques remains to be established in prospective, controlled studies with longer follow-up.

## 1. Introduction

Anterior cruciate ligament (ACL) rupture represents one of the most prevalent ligamentous injuries affecting the knee joint, with an annual incidence approaching 75 per 100,000 individuals in the general population [1]. This injury predominantly affects young, physically active individuals engaged in contact and pivoting sports, with a notable increase in pediatric populations attributed to earlier sports participation [2]. The female athletic population demonstrates a disproportionately higher injury rate per athletic exposure compared to males [3,4].

The inherent limited biological healing capacity of the ACL necessitates surgical reconstruction to restore functional joint stability and prevent the accelerated development of post-traumatic osteoarthritis [5,6]. Consequently, ACL reconstruction has become one of the most frequently performed orthopedic procedures, with surgical volume increasing by approximately 37% in recent years [7].

The limited intrinsic healing capacity of the ACL, in contrast to the favorable healing potential of the medial collateral ligament (MCL), has increasingly been attributed to differences in the epiligament, the thin, vascularised connective tissue layer that ensheathes ligaments and harbors stem-cell-like, neural and vascular elements critical for ligament repair. Recent histological and molecular investigations (2024–2026) have demonstrated that the ACL epiligament is thinner, less cellular, and less vascularised than that of the MCL, with regional differences between the proximal (femoral), mid-substance, and distal (tibial) portions of the ACL [8,9]. These regional variations in epiligament thickness, vascular density, and progenitor-cell content provide a biological explanation for the well-documented failure of primary ACL healing and support the rationale for reconstruction rather than primary repair in the majority of cases [10]. An appreciation of epiligament biology is also relevant to current reconstructive strategies, since preservation of the remnant and its epiligament sleeve during tunnel creation may influence graft vascularisation, proprioceptive reinnervation and ligamentisation.

Arthroscopic ACL reconstruction has emerged as the standard of care, offering a minimally invasive approach to achieving anatomic ligament restoration [11]. Non-anatomic reconstructions have been associated with persistent rotational instability, graft attenuation, and impingement phenomena [12,13]. Current evidence emphasizes the importance of anatomic tunnel placement to replicate the native ACL footprint and restore physiological knee kinematics.

Two predominant techniques for femoral tunnel creation have gained widespread acceptance: the transportal and all-inside techniques (AIT). The transportal approach facilitates independent femoral tunnel drilling and offers technical accessibility without requiring specialized instrumentation [14,15,16]. The all-inside technique, introduced as an alternative, utilizes socket-type tunnels that potentially reduce postoperative pain, effusion, and synovial fluid extravasation at the graft-bone interface [17,18,19]. Advantages of AIT include smaller incisions, reduced invasiveness, and versatility in graft options [20,21].

The far anteromedial portal technique represents a refinement of the transportal approach, enabling optimal visualization and accurate anatomic tunnel positioning within the native ACL footprint. Combined with retrograde femoral socket drilling, this technique may theoretically optimize tunnel orientation and graft positioning while minimizing soft tissue trauma. Although the term “retrograde drilling” has historically been associated with all-inside reconstructions, in the present technique it refers specifically to the use of a retrograde cutter (FlipCutter) introduced through a far anteromedial portal to create a closed-socket femoral tunnel, while the tibial tunnel is made in a conventional antegrade, outside-in fashion (i.e., this is not an “all-inside” reconstruction).

The objective of this study was to descriptively evaluate the early functional outcomes of anatomic ACL reconstruction performed using retrograde femoral socket drilling and the far anteromedial portal technique with hamstring tendon autograft. We hypothesized that this technique would be associated with satisfactory functional improvement as measured by validated patient-reported outcome instruments, comparable to outcomes previously reported for contemporary anatomic ACL reconstruction. The study was not designed to demonstrate superiority over other techniques.

## 2. Materials and Methods

This observational study is reported in accordance with the STROBE (Strengthening the Reporting of Observational Studies in Epidemiology) recommendations [22].

### 2.1. Study Design and Patient Population

This retrospective cohort study was conducted at the Department of Orthopedics and Traumatology, İnönü University Faculty of Medicine, between January 2021 and December 2021. The study protocol was approved by the institutional ethics committee, and all patients provided written informed consent prior to enrollment.

Inclusion criteria comprised: (1) patients aged 18–50 years with complete ACL rupture confirmed by clinical examination and magnetic resonance imaging (MRI); (2) primary ACL reconstruction using hamstring tendon autograft; and (3) minimum 12-month postoperative follow-up. Exclusion criteria included: (1) multi-ligamentous knee injuries requiring concurrent reconstruction; (2) revision ACL surgery; (3) contralateral ACL deficiency; (4) inflammatory arthropathy or systemic connective tissue disorders; and (5) previous ipsilateral knee surgery.

A total of 33 consecutive patients meeting the eligibility criteria were enrolled in the study. During the study period, 52 patients undergoing primary ACL reconstruction were screened: 10 were excluded because of multi-ligamentous injury requiring concurrent reconstruction, 4 for revision ACL surgery, 2 for contralateral ACL deficiency, 2 for previous ipsilateral knee surgery, and 1 for an inflammatory arthropathy, leaving 33 eligible patients, all of whom completed 12-month follow-up (no patients were lost to follow-up; there were no missing data for the primary or secondary outcomes, so no imputation was required). All patients underwent comprehensive preoperative clinical and radiological evaluation, including standardized physical examination (Lachman test, anterior drawer test, pivot shift test), plain radiographs, and MRI of the affected knee.

### 2.2. Surgical Technique

**Figure 1:**
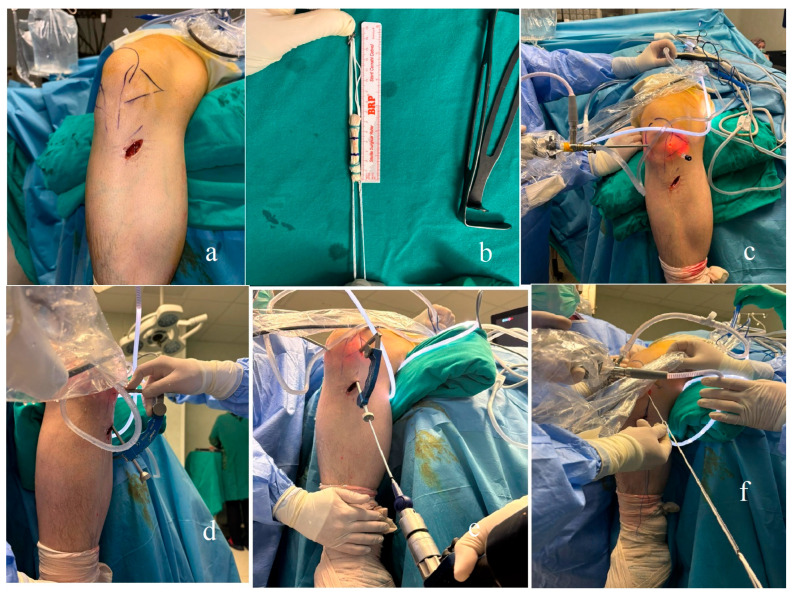
Intraoperative surgical photographs. Graft donor site(**a**), preparation of the harvested graft, with a single tendon quadrupled (**b**), portal sites and the extra-far medial portal (**c**), tibial guide and preparation of the tibial tunnel (**d**,**e**), passing the tendon graft through the medial portal prior to fixation (**f**).

**Figure 2:**
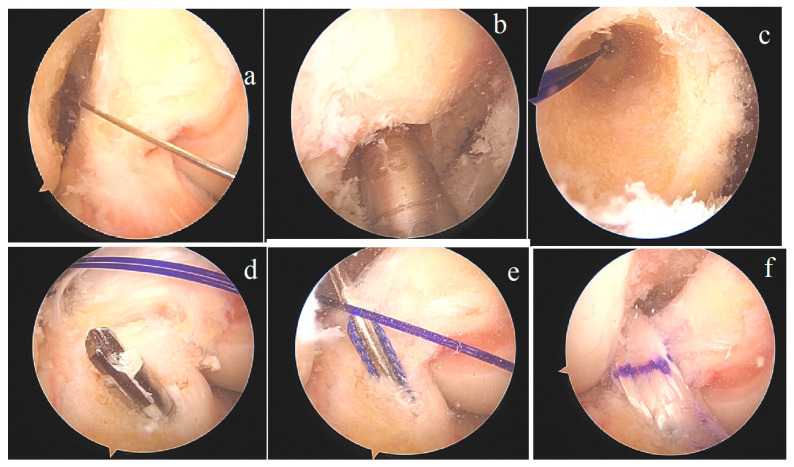
Intraoperative arthroscopic views. Ruptured ACL and the location of the femoral tunnel (**a**), opening of the femoral tunnel (**b**) and view from within the far medial portal (**c**), open tibial tunnel and ACL reconstruction (**d**–**f**).

All surgical procedures were performed by a single senior orthopedic surgeon (E.E.) under general or regional anesthesia with the patient positioned supine on a standard operating table with a lateral thigh post and foot support to maintain knee flexion (Figure 1 and Figure 2). In the present technique, only the femoral socket is created with a retrograde cutter (FlipCutter, Arthrex Inc., Naples, FL, USA) introduced through a far anteromedial portal, while a full tibial tunnel is drilled in a conventional antegrade, outside-in fashion with a standard aiming guide. This combination differs from the classical anteromedial transportal technique, in which the femoral tunnel is drilled antegrade through the medial portal, and from all-inside reconstructions, in which both the femoral and tibial tunnels are created as closed sockets with retrograde cutters. The key technical novelty is therefore the combination of a retrograde, closed-socket femoral tunnel drilled through an extra-far (inferomedial) anteromedial portal with a conventional full tibial tunnel, aiming to improve the obliquity and anatomical position of the femoral tunnel while avoiding the trans-tibial constraints and the fully socketed tibial fixation of all-inside techniques.

Graft Harvesting: The semitendinosus tendon was harvested through an oblique or longitudinal incision (4–5 cm) placed two fingerbreadths medial and one fingerbreadth superior to the tibial tubercle, extending distally. Following skin and subcutaneous dissection, the pes anserinus fascia was identified and incised in an “L-shaped” configuration. The semitendinosus tendon was isolated, marked with suture, and harvested using a tendon stripper directed toward the ischial tuberosity. The harvested tendon was prepared on a back table, with meticulous removal of muscle tissue and whip-stitching of the free ends. The graft was quadrupled and tensioned on a graft preparation board. Final graft diameter was measured using sizing cylinders.

Portal Establishment and Diagnostic Arthroscopy: Standard arthroscopic portals were established. A high anterolateral portal was created adjacent to the lateral border of the patellar tendon. A far (extra-far) anteromedial portal was established approximately 1 cm above the medial joint line and as medial as possible to allow an oblique trajectory toward the lateral femoral condyle to provide anterior orientation for femoral tunnel drilling. Portal placement was confirmed arthroscopically using a spinal needle. Systematic diagnostic arthroscopy was performed to assess meniscal and chondral pathology prior to reconstruction.

Femoral Tunnel Preparation: With the knee positioned in approximately 110–120 degrees of hyperflexion, the center of the femoral footprint was marked using an awl or curette. The femoral socket was created using a retrograde drilling system (FlipCutter, Arthrex Inc., Naples, FL, USA) introduced through the far anteromedial portal. The socket was drilled to a depth of 20–25 mm with a diameter matching the prepared graft.

Tibial Tunnel Preparation: The tibial tunnel was created using an outside-in technique with a tibial aiming guide set at 55 degrees. The guide was positioned to target the center of the native ACL tibial footprint, verified arthroscopically.

Graft Passage and Fixation: The quadrupled semitendinosus graft was passed through the tibial tunnel and into the femoral socket using passing sutures. Femoral fixation was achieved using a cortical suspensory device (EndoButton CL, Smith & Nephew, Andover, MA, USA). The graft was tensioned with the knee in 20–30 degrees of flexion, and tibial fixation was performed using an interference screw supplemented with a cortical button or staple as needed.

### 2.3. Postoperative Rehabilitation

A standardized four-phase rehabilitation protocol was implemented for all patients. Progression between phases was criterion-based rather than strictly time-based and required attainment of the milestones specified below.

Phase I (Days 0–7): Emphasis on pain and effusion management, passive range of motion exercises targeting full extension and 90 degrees of flexion using continuous passive motion (CPM), quadriceps isometric exercises, and progression to straight leg raises.

Phase II (Week 2–4): Achievement of full extension, gradual restoration of full flexion range, patellar mobilization exercises, progressive hamstring strengthening, and initiation of closed kinetic chain exercises (partial weight-bearing mini-squats to 45° and leg press to 60°). Progression to Phase III required full passive extension, flexion ≥ 120°, absence of effusion, and the ability to perform a unilateral straight-leg raise without extension lag.

Phase III (Weeks 5–8): Continuation of previous exercises with addition of active knee extension (90–30 degrees), lateral step-ups, and progression of closed kinetic chain exercises (mini-squats to 60°, leg press to 90°, step-ups, and stationary cycling). Proprioceptive training was introduced progressively from double-leg to single-leg stance exercises on stable and unstable surfaces. Progression to Phase IV required pain-free full range of motion, symmetrical gait, and quadriceps strength ≥60% of the contralateral limb on manual or isokinetic testing.

Phase IV (Week 8 onwards): Proprioceptive training, arthrometric stability assessment at 10 weeks, isokinetic testing, hop testing at 12 weeks, light jogging when quadriceps strength reaches 70% of the contralateral limb, and sport-specific exercises when strength reaches 80%. Return-to-running criteria included ≥70% quadriceps limb symmetry index, no effusion, and full range of motion; return-to-sport decisions required ≥90% limb symmetry on isokinetic and hop testing batteries, absence of pain or effusion, clinical stability on Lachman and pivot-shift testing, and psychological readiness. Return-to-sport rates and timing were not systematically collected in the present study and are therefore not reported.

### 2.4. Outcome Assessment

The pre-specified primary outcome was the change in the Tegner-Lysholm Knee Scoring Scale score between the preoperative assessment and the 12-month postoperative assessment. Pre-specified secondary outcomes were the Modified Cincinnati Rating System score, the IKDC subjective knee evaluation form score, and the results of the Lachman, anterior drawer and pivot-shift clinical stability tests. Objective instrumented laxity measurement (e.g., KT-1000/2000, MEDmetric, San Diego, CA, USA) was not routinely available at our institution during the study period and was therefore not included as an outcome.

Functional outcomes were evaluated using three validated patient-reported outcome instruments administered preoperatively and at 6 weeks, 3 months, and 12 months postoperatively:

1. Tegner-Lysholm Knee Scoring Scale: A 100-point scale assessing symptoms and functional limitations, with scores categorized as excellent (95–100), good (84–94), fair (65–83), or poor (<65).
2. Modified Cincinnati Rating System: A comprehensive knee rating system evaluating symptoms, functional capacity, and activity level, with scores interpreted as excellent (>80), good (55–79), fair (30–54), or poor (<30).
3. International Knee Documentation Committee (IKDC) Subjective Knee Evaluation Form: An 18-item instrument assessing symptoms, sports activities, and knee function, with higher scores indicating better function.

Clinical examination including the Lachman test, anterior drawer test, and pivot shift test was performed preoperatively and at final follow-up, with findings documented.

### 2.5. Statistical Analysis

Statistical analyses were performed using SPSS version 25.0 (IBM Corp., Armonk, NY, USA). Continuous variables were expressed as mean ± standard deviation with range and 95% confidence intervals for mean changes, and categorical variables were presented as frequencies and percentages. The Shapiro–Wilk test was used to assess normality of data distribution.

Correlations between continuous variables were analyzed using Spearman’s rank correlation coefficient. Comparisons between groups were performed using the Mann–Whitney U test for two independent groups and the Kruskal–Wallis H test for multiple groups. Changes in functional scores across the four time points (preoperative, 6 weeks, 3 months, 12 months) were analyzed using the Friedman test for repeated measures on non-normally distributed data, with post hoc pairwise comparisons performed using the Wilcoxon signed-rank test and Bonferroni correction for multiple comparisons. Changes in categorical stability findings (pre- vs.post-operative) were assessed with McNemar’s test. Effect sizes for paired changes were calculated as matched-pairs r values and are reported alongside the corresponding test statistics in the Section 3. Observed mean changes were compared against previously published minimal clinically important differences (MCID) for the Lysholm, IKDC and Cincinnati scores in ACL populations. No multivariable or propensity-adjusted models were used because of the modest sample size; accordingly, all subgroup analyses (acute vs.chronic, sports vs. non-sports, with vs. without concomitant pathology) and correlation analyses involving the interval from injury to surgery were considered exploratory and hypothesis-generating and should not be interpreted as evidence of causal effects. A two-tailed *p*-value < 0.05 was considered statistically significant.

## 3. Results

### 3.1. Patient Demographics and Injury Characteristics

A total of 33 patients were enrolled and completed the study protocol. Patient demographics and injury characteristics are summarized in Table 1. All patients were male, with a mean age of 28 years (range: 18–44 years). The all-male composition of the cohort is a structural limitation that must be considered when interpreting all subsequent results and that markedly limits external validity to female patients, who represent a large proportion of the ACL-injured population. The predominant mechanism of injury was sports-related in 28 patients (84.8%), while 5 patients (15.2%) sustained injuries through other mechanisms. Right knee involvement was observed in 21 cases (63.6%) and left knee in 12 cases (36.4%).

The mean interval from injury to surgical reconstruction was 9.5 months (range: 0.25–120 months). For the purpose of exploratory subgroup analyses, patients were pre-specified as having an “acute” presentation if surgery occurred within 3 months of the index injury and as having “chronic” ACL deficiency if surgery was performed >3 months after injury, in line with commonly used thresholds in the ACL reconstruction literature. Fifteen patients (45.5%) were classified as acute presentations (surgery within 3 months of injury), while 18 patients (54.5%) presented with chronic ACL deficiency.

The mean graft diameter achieved was 8.4 mm (range: 7–9 mm). Concomitant intra-articular pathology was identified in 17 patients: isolated meniscal lesions in 10 patients (30.3%), osteochondral defects (OCD) in 3 patients (9.1%), and combined meniscal and osteochondral pathology in 4 patients (12.1%). The remaining 16 patients (48.5%) had isolated ACL rupture without associated pathology.

The mean follow-up duration was 17 months (range: 13–24 months). No patients were lost to follow-up, and there were no cases of graft failure, deep infection, or major complications requiring reoperation.

### 3.2. Preoperative Clinical Findings

Preoperative clinical examination demonstrated positive Lachman test in 30 patients (90.9%): grade I in 26 patients (78.8%) and grade II in 4 patients (12.1%). The pivot shift test was positive in 21 patients (63.6%), with grade I in 12 (36.4%), grade II in 8 (24.2%), and grade III in 1 (3.0%)(Table 2).

Preoperative functional scores reflected significant disability. The mean Tegner-Lysholm score was 46.8 ± 17.3 (range: 21–86; median: 50), indicating poor to fair knee function. The mean IKDC score was 36.3 ± 14.4 (range: 16.1–81.6; median: 34.5), and the mean Cincinnati score was 41.3 ± 15.9 (range: 12–85; median: 42).

Significant positive correlations were observed between all three preoperative functional scores (Spearman r = 0.57–0.60, all *p* < 0.01). Because the Tegner-Lysholm, IKDC and Cincinnati instruments are designed to measure overlapping constructs of subjective knee function, these inter-instrument correlations are expected and are noted for completeness rather than interpreted as independent findings; accordingly, they are not reiterated at each time point.

### 3.3. Postoperative Functional Outcomes

Substantial improvement in all functional outcome measures was observed following ACL reconstruction. The comparative analysis of preoperative and postoperative functional scores is presented in Table 3. The Friedman test confirmed a significant overall effect of time for each of the three instruments (*p* < 0.001), and all pairwise post hoc Wilcoxon comparisons between time points remained significant after Bonferroni correction. Matched-pairs effect sizes were large for all three instruments (Tegner-Lysholm r = 0.87; IKDC r = 0.84; Cincinnati r = 0.86), and the magnitude of the 12-month change for each instrument exceeded commonly cited MCID estimates (Lysholm ≈ 10 points; IKDC ≈ 11 points; Cincinnati ≈ 10–12 points), as detailed in Table 3, confirming both the statistical and clinical relevance of the observed improvements.

Analysis of temporal progression demonstrated consistent improvement across all assessment intervals. The Tegner-Lysholm score increased from 46.8 ± 17.3 preoperatively to 65.3 ± 18.6 at 6 weeks, 75.3 ± 14.4 at 3 months, and 83.7 ± 10.5 at 12 months (mean change 0–12 months +36.9, 95% CI 30.3 to 43.5). The corresponding mean changes were +32.1 (95% CI 25.3 to 38.9) for the IKDC score and +38.9 (95% CI 32.6 to 45.2) for the Cincinnati score. Strong positive correlations were observed between scores at successive time points (6-week to 3-month: r = 0.667, *p* < 0.01; 3-month to 12-month: r = 0.645, *p* < 0.01; 6-week to 12-month: r = 0.528, *p* < 0.01).

Postoperative cross-instrument correlations remained strong (Spearman r = 0.56–0.71, all *p* < 0.01), consistent with the expected construct overlap noted in Section 3.2.

### 3.4. Subgroup Analyses

Subgroup analyses were performed to evaluate the influence of patient and injury characteristics on functional outcomes. All subgroup comparisons presented below are exploratory, were unadjusted for potential confounders (including baseline activity level, concomitant meniscal or osteochondral procedures, rehabilitation adherence and injury chronicity), and are limited by small subgroup sizes (≤18 per group). They should therefore be regarded as hypothesis-generating rather than as definitive evidence of associations.

Timing of Surgery: No statistically significant differences in preoperative or postoperative functional scores were observed between patients with acute versus chronic ACL deficiency (*p* > 0.05 for all comparisons).

Mechanism of Injury: Comparison between sports-related and non-sports-related injuries revealed no significant differences in any functional outcome measure (*p* > 0.05 for all comparisons).

Concomitant Pathology: The presence of associated meniscal or osteochondral pathology did not significantly affect preoperative or postoperative functional scores (*p* > 0.05 for all comparisons), although a trend toward lower postoperative Cincinnati scores was observed in patients with combined pathology.

Interval from Injury to Surgery: A statistically significant but exploratory positive correlation was identified between the time from ACL rupture to surgical reconstruction and postoperative Tegner-Lysholm score (r = 0.486, *p* < 0.01) and Cincinnati score (r = 0.429, *p* < 0.05). This correlation is highly susceptible to confounding, including selection of more active patients for earlier surgery, differences in preoperative rehabilitation, persistent effusion or reduced range of motion at the time of early surgery, and small-sample instability, and was not adjusted in any multivariable model. It should therefore not be interpreted as evidence that delayed reconstruction causes better functional outcomes, suggesting that delayed reconstruction did not appear to adversely affect early functional outcomes in this cohort.

### 3.5. Postoperative Clinical Examination and Stability

At final follow-up, clinical examination demonstrated restoration of knee stability in the majority of patients, as detailed in Table 4. The anterior drawer test was negative in 22 patients (66.7%) and graded I (0–5 mm) in 11 patients (33.3%). No patients demonstrated grade II or III anterior drawer instability. The Lachman test was negative in 27 patients (81.8%) and grade I in 6 patients (18.2%).

Clinically suspected (i.e., not radiologically confirmed) femoral-sided graft loosening was documented on examination in 4 patients (12.1%), while clinically suspected tibial-sided loosening was observed in 6 patients (18.2%). In the absence of routine postoperative MRI or instrumented laxity testing, these findings represent a clinical impression rather than a confirmed structural diagnosis. However, these findings did not significantly correlate with postoperative functional scores (*p* > 0.05 for all comparisons), although a non-significant trend toward lower IKDC scores was observed in patients with suspected femoral loosening (mean rank: 9.0 vs. 18.1; *p* = 0.07), which should be interpreted cautiously given the very small subgroup size (n = 4) and the absence of multivariable adjustment.

No major complications were observed during the study period. There were no cases of graft failure, deep infection, arthrofibrosis, or other complications requiring reoperation.

## 4. Discussion

The principal finding of this study is that anatomic ACL reconstruction utilizing retrograde femoral-socket drilling and the far anteromedial portal technique with hamstring tendon autograft was associated with satisfactory early functional outcomes in a small, homogeneous all-male cohort. Significant improvements were demonstrated across all validated patient-reported outcome measures, with the majority of patients achieving good to excellent functional status at 12-month follow-up. Because the study had no concurrent comparator, no multivariable adjustment and a small, all-male sample, these observations should be interpreted descriptively and should not be read as evidence of technique-specific superiority.

The surgical technique employed in this study combines several potentially advantageous features of contemporary ACL reconstruction approaches. The far anteromedial portal provides optimal visualization of the femoral footprint and facilitates accurate anatomic tunnel placement, which has been established as a critical determinant of successful outcomes [23,24]. The retrograde tibial drilling technique offers a less invasive approach to femoral tunnel creation while maintaining the ability to achieve anatomic positioning [25]. Because the present study did not include a concurrent comparator group, however, these features remain theoretical advantages at the cohort level rather than demonstrated benefits.

From a biological standpoint, the limited intrinsic healing capacity of the ACL, which mandates reconstruction rather than primary repair in most clinical scenarios, is increasingly understood in terms of epiligament structure and function. Recent work from 2024 to 2026 has demonstrated that the ACL epiligament is thinner, less cellular and less vascularised than that of the MCL, and that regional variations exist along the femoral, mid-substance and tibial portions of the ACL, with the mid-substance region appearing particularly hypocellular and hypovascular [8,9,10]. These differences help to explain why MCL tears heal reliably after conservative treatment whereas ACL tears rarely achieve functional healing, and they have direct implications for reconstructive strategy: maximal preservation of the native ACL remnant and its epiligament sleeve during notch preparation may contribute to graft revascularisation, proprioceptive reinnervation and ligamentisation. The present technique, which relies on a retrograde femoral cutter introduced through a far anteromedial portal, permits socket creation with limited disruption of the surrounding soft tissue envelope and is in principle compatible with a remnant-preserving approach; whether this translates into improved biological integration, however, cannot be inferred from the present functional data and would require histological, imaging or second-look arthroscopic evaluation in future work.

Our results are consistent with previously published outcomes of anatomic ACL reconstruction techniques. The mean postoperative Tegner-Lysholm score of 83.7 compares favorably with scores reported in the literature, which typically range from 85 to 95 for successful reconstructions [26,27,28]. Similarly, the IKDC scores and Cincinnati scores achieved in our cohort fall within the expected range for satisfactory outcomes following ACL reconstruction [29,30]. Given that the observed outcomes are within the reported range for contemporary anatomic reconstructions in general, they should be interpreted as consistent with, rather than superior to, existing techniques.

The all-male composition of our study cohort reflects the epidemiology of ACL injuries in our regional population and the predominance of contact sports participation among males. While this limits the generalizability of our findings markedly to female populations, it provides a homogeneous sample for evaluating surgical outcomes. Given the well-recognized differences in ACL injury mechanics, hormonal milieu, neuromuscular control and post-reconstruction outcomes between sexes, the present findings cannot be extrapolated to female patients, and this should be viewed as a major structural limitation rather than a neutral feature of the cohort. The high proportion of sports-related injuries, which accounted for the vast majority of cases, is consistent with the typical mechanism of ACL rupture and supports the relevance of our findings to the athletic population.

An exploratory observation from our subgroup analyses was the positive correlation between the interval from injury to surgery and postoperative functional scores. This finding is subject to substantial confounding—including selection of more active or better-rehabilitated patients for later surgery, resolution of effusion and restoration of motion prior to delayed reconstruction, and small-sample instability—and it contrasts with the conventional assumption that early reconstruction uniformly yields superior outcomes, but it cannot establish a causal effect of delay on outcomes. It suggests that patient selection and appropriate timing may be more nuanced than previously appreciated. Delayed reconstruction may allow resolution of acute inflammation and restoration of range of motion, potentially optimizing the surgical environment and rehabilitation course [31]; however, a prospective, adjusted analysis would be required to confirm this.

The utilization of semitendinosus-only autograft in our technique represents a potential advantage in terms of donor site morbidity. Preservation of the gracilis tendon may maintain hamstring strength and reduce the risk of flexion weakness, which has been reported as a concern with combined semitendinosus-gracilis harvest [32,33]. The mean graft diameter of 8.4 mm achieved in our series is within the acceptable range for adequate mechanical strength, as graft diameters below 7–8 mm have been associated with increased failure rates [34,35].

The retrograde drilling technique employed in this study requires hyperflexion of the knee for femoral tunnel creation, which represents a technical consideration and potential limitation. This position may be challenging in patients with limited preoperative range of motion or those under regional anesthesia. However, the technique offers the advantage of independent tunnel drilling without the need for a trans-tibial approach, which has been associated with non-anatomic tunnel placement [36,37].

The postoperative stability results demonstrate excellent restoration of knee stability, with more than two-thirds of the patients achieving a negative anterior drawer test at the final follow-up. The absence of major complications, including graft failure and deep infection, further supports the safety profile of this technique.

Several limitations of this study warrant acknowledgment. First, the retrospective design introduces inherent biases related to data collection and patient selection. Second, the single-arm design without a concurrent comparator (e.g., standard anteromedial transportal reconstruction or all-inside reconstruction) precludes any assessment of comparative effectiveness and means that the observed outcomes cannot be causally attributed to the specific combination of retrograde femoral socket drilling and the far anteromedial portal beyond what would be expected from contemporary anatomic ACL reconstruction in general. Third, no multivariable models were used to adjust for potential confounders such as injury chronicity, concomitant meniscal or osteochondral procedures, baseline activity level and rehabilitation adherence; all subgroup and correlation analyses are therefore exploratory. Fourth, the modest sample size limits the generalizability and statistical power of our findings, particularly for subgroup comparisons. Fifth, the absence of a control group precludes direct comparison with alternative surgical techniques. Sixth, the follow-up duration, while adequate for early outcome assessment, does not capture long-term results including potential graft failure at 2–10 years, return-to-sport rates, or development of post-traumatic osteoarthritis. Seventh, objective instrumented laxity (e.g., KT-1000/2000) and systematic return-to-sport data were not collected, which limits direct comparison with the sports-medicine and physiotherapy literature. Eighth, clinically suspected femoral or tibial graft loosening was not confirmed with postoperative MRI, CT or instrumented laxity measurement and therefore represents a clinical impression only. Ninth, the all-male cohort limits applicability to female patients, who represent a significant proportion of ACL-injured individuals.

The strengths of this study include the standardized surgical technique performed by a single experienced surgeon, consistent measurement using multiple validated patient-reported outcome instruments in a single-surgeon series, and complete follow-up at all time points. The use of three complementary functional scores provides a comprehensive evaluation of patient-reported outcomes that, together with the modest sample size and observational design, supports the descriptive conclusions drawn.

Future investigations should include prospective cohort studies and randomized controlled trials comparing this technique with established approaches such as standard transportal and all-inside techniques, ideally with pre-specified multivariable adjustment, sex-balanced enrolment, and the inclusion of both subjective and objective outcomes (instrumented laxity, return-to-sport rates, revision rates and radiographic osteoarthritis). Long-term follow-up studies are essential to evaluate graft survival, return-to-sport outcomes, and the incidence of post-traumatic osteoarthritis. Biomechanical studies comparing tunnel orientation and graft positioning between techniques would provide valuable mechanistic insights. Integration of epiligament-oriented biological endpoints (e.g., remnant preservation, graft vascularisation and ligamentisation on sequential MRI) in future trials could help translate the recent biological literature on ACL healing into reconstructive practice.

## 5. Conclusions

Anatomic ACL reconstruction using retrograde femoral socket drilling using a FlipCutter through a far anteromedial portal combined with a conventional antegrade tibial tunnel with semitendinosus autograft was associated with satisfactory early functional outcomes in a small, all-male cohort, comparable to those reported for contemporary anatomic ACL reconstruction techniques, as evidenced by significant improvements in all validated outcome measures. This technique offers several potential advantages, including reduced donor site morbidity through single-tendon harvest, facilitation of anatomic tunnel placement, and adequate restoration of knee stability. The requirement for knee hyperflexion during femoral tunnel drilling represents a technical consideration. Because the present study is a retrospective, single-arm cohort without a concurrent comparator, without multivariable adjustment and without objective laxity or return-to-sport data, these findings should be regarded as descriptive and hypothesis-generating and do not establish superiority over alternative anatomic techniques. The comparative effectiveness of this technique relative to standard anteromedial transportal and all-inside reconstructions remains to be defined in prospective, controlled studies with longer follow-up and objective outcome measures. Randomized controlled trials with extended follow-up and objective outcome measures are warranted to establish the comparative effectiveness of this technique and define its optimal indications in the spectrum of ACL reconstruction approaches.

## Figures and Tables

**Table 1:** Patient Demographics and Injury Characteristics (*n* = 33).

Variable	Value
Demographic Characteristics	
Age, years (mean ± SD)	28 ± 7.2 (range: 18–44)
Sex, male *n* (%)	33 (100)
Injury Characteristics	
Mechanism of injury, n (%)	
Sports-related	28 (84.8)
Other	5 (15.2)
Affected side, *n* (%)	
Right knee	21 (63.6)
Left knee	12 (36.4)
Time from injury to surgery, months (mean ± SD)	9.5 ± 22.1 (range: 0.25–120)
Injury timing, *n* (%)	
Acute (≤3 months)	15 (45.5)
Chronic (>3 months)	18 (54.5)
Concomitant Pathology	
Isolated ACL rupture, *n* (%)	16 (48.5)
Meniscal lesion, *n* (%)	10 (30.3)
Osteochondral defect (OCD), *n* (%)	3 (9.1)
Meniscus + OCD, *n* (%)	4 (12.1)
Graft Characteristics	
Graft diameter, mm (mean ± SD)	8.4 ± 0.6 (range: 7–9)
Follow-up	
Follow-up duration, months (mean ± SD)	17 ± 3.4 (range: 13–24)
Lost to follow-up, *n* (%)	0 (0)

Abbreviations: ACL, anterior cruciate ligament; OCD, osteochondral defect; SD, standard deviation.

**Table 2:** Preoperative Clinical Examination Findings (*n* = 33).

Clinical Test	Grade	*n* (%)	Total Positive
Lachman Test			30 (90.9%)
	Negative	3 (9.1)	
	Grade I (+)	26 (78.8)	
	Grade II (++)	4 (12.1)	
	Grade III (+++)	0 (0)	
Pivot Shift Test			21 (63.6%)
	Negative	12 (36.4)	
	Grade I (Glide)	12 (36.4)	
	Grade II (Clunk)	8 (24.2)	
	Grade III (Gross)	1 (3.0)	
Anterior Drawer Test			32 (97.0%)
	Negative	1 (3.0)	
	Grade I (0–5 mm)	16 (48.5)	
	Grade II (5–10 mm)	11 (33.3)	
	Grade III (>10 mm)	5 (15.2)	

Grading based on International Knee Documentation Committee criteria. Lachman test grading: Grade I, 3–5 mm; Grade II, 5–10 mm; Grade III, >10 mm anterior translation.

**Table 3:** Functional Score Comparison: Preoperative vs.12-Month Postoperative (*n* = 33).

Score	Preoperative Mean ± SD	Postoperative (12 Months) Mean ± SD	Mean Change	95% CI	Effect Size r	*p*-Value
Tegner-Lysholm	46.8 ± 17.3	83.7 ± 10.5	+36.9	30.3 to 43.5	0.87	<0.001 *
IKDC	36.3 ± 14.4	68.4 ± 15.1	+32.1	25.3 to 38.9	0.84	<0.001 *
Cincinnati	41.3 ± 15.9	80.2 ± 10.9	+38.9	32.6 to 45.2	0.86	<0.001 *

Abbreviations: IKDC, International Knee Documentation Committee; SD, standard deviation; CI, confidence interval. Effect size r = matched-pairs rank-biserial correlation (Wilcoxon signed-rank test). * Statistically significant (*p* < 0.05). Statistical analysis performed using the Friedman test with post hoc Wilcoxon signed-rank tests and Bonferroni correction.

**Table 4:** Postoperative Stability Assessment and Complications (*n* = 33).

Parameter	Preoperative *n* (%)	Postoperative *n* (%)	*p*-Value
Anterior Drawer Test			<0.001 *
Negative	1 (3.0)	22 (66.7)	
Grade I (0–5 mm)	16 (48.5)	11 (33.3)	
Grade II (5–10 mm)	11 (33.3)	0 (0)	
Grade III (>10 mm)	5 (15.2)	0 (0)	
Lachman Test (Postoperative)			-
Negative	-	27 (81.8)	
Grade I	-	6 (18.2)	
Grade II-III	-	0 (0)	
Clinically suspected graft loosening			
Femoral side	-	4 (12.1)	-
Tibial side	-	6 (18.2)	-
Complications			
Graft failure/re-rupture	-	0 (0)	-
Deep infection	-	0 (0)	-
Arthrofibrosis	-	0 (0)	-
Reoperation required	-	0 (0)	-

* Statisticallysignificant (*p* < 0.05). Statistical analysis performed using McNemar’s test for categorical variables. Graft loosening entries refer to clinically suspected loosening on examination and were not confirmed by postoperative imaging or instrumented laxity measurement.

## Data Availability

The data presented in this study are available on request from thecorresponding author due to the detailed and sensitive personal information of the participants.
